# Anecdote of Thomas Paine

**Published:** 1866-11

**Authors:** 


### ANECDOTE OF THOMAS PAINE.

In 1802, I boarded at the same house, and ate at the same table, with Thomas Paine. From his dissipated habits, slovenly apparel, and filthy person, he was shunned by all the superfine-coat infidels in town and country. He boarded in the house of William Carver, a journeyman blacksmith; his chief companions were journeymen mechanics. Generally, twelve or fifteen assembled in his room every evening; to them he preached the cold and cheerless doctrines of infidelity. Many of them left the church, spent their wages in grog-cellars, became drunkards, died beggars, while their widows and orphans slept in almshouses.

One evening, I entered his room and sat down; he was accusing the Bible and its friends, as being the cause of all the bloodshed and mischief in the world, since the days of Noah's flood. He ceased. Said I: "Mr. Paine, you have been in France, Spain, Rome, Ireland. You find no bibles there: granted. There, the common people are ignorant and brutish, live in hovels, like beasts that perish: granted. You have been in Scotland, where every house and cottage has its half a dozen bibles." "Its true," said he, "and they are the most superstitious bigots in the world." Now, says I, "Mr. Paine, if the bible was a bad book, those who use it most would be the worst members of society; but, as it regards Scotchmen, the reverse is the fact. Yesterday's journal contained a list of the inmates of the State-prison, Penitentiary, and Almshouse, with the name of the countries whence they came, and excepting four Englishmen, they were all from countries, where they never saw the bible; but there was not a Scotchman, woman or child, among them."

At this moment the clock struck ten, he lifted a candle from the table, and without speaking, went up to bed, leaving his friends and myself to draw our own conclusions. Grant Thorburn.

In a private note, our venerable correspondent, in his eighty-seventh year, said, that he and Paine boarded with Carver and his wife, both of whom, with Paine, were from the same town in England. "We used to sit by Carver's fire of nights, while I heard them live their lives over again; thus, I learned his life from the cradle, till I followed him to his grave, in 1809."

THAT able scholar and gifted divine, W. A. Scott, D.D., some time ago has issued another 12mo., of 353 pp., through his publishers, H. H. Bancroft & Co., of San Francisco. The mechanical getting up, both as to type and paper, would be creditable to New York, while the subject matter, and the mode of handling it, are well worthy of high praise. We knew Dr. Scott to be a Bible preacher, many years ago, in the Crescent City, and it was to that he owed his power over the people, his crowded houses, and his extended fame; hence, it is no wonder that, in his writings, the Bible and its illustration and exposition are his Alpha and Omega, the beginning and the end and the middle of every book, as their titles indicate: 1. Daniel, a Model for Young Men; 2nd. The Wedge of Gold; or, Achan in El Dorado; 3rd. Trade and Letters: their Journeyings Round the World; 4th. The Giant Judge; or, Samson, the Hebrew Hercules; and this last, Esther, the Hebrew Queen, the introduction to which ought to be made a tract of, and scattered by millions on the wings of every wind, throwing out, as it does, deep thoughts to clergymen, to mothers and daughters, to the Press, and to Christians of every name, as also to errorists, fanatics, and rationalists, in that all should take the whole Bible as their guide, looking for no new or other revelations, neither by vision, nor voices, nor dreams, nor angels, nor spirits, nor internal illumination, neither to add to, nor even explain the Bible. He is more and more convinced, that one great cause of the modern growth of fanaticism and infidelity, is to be found in the departure of so many teachers from the custom of reading and expounding the Word of God. That it is worthy of serious consideration, whether there is not, and to what extent in our day, in the topical, metaphysical preaching of many, and in not a few of our popular tracts and treatises on practical and experimental religion, theological essays, religious essays, and pious novels, which are worse than the pious frauds of the dark ages, a dangerous tendency to draw away the public mind from the Book of God—whether the frequent religious meetings, the cramming of the Lord's Day, and the tendency of the popular religious literature of our time, is not towards a substituting of tracts, and books, and newspapers, *about* religion, for the Book of the Lord. There is no hand-book for revivals like the inspired history of remarkable Bible conversions. For family reading and catechising on the afternoon on the Lord's Day, the hot-house system, now so much in vogue, is a poor substitute. For the family, and the place of business, the church, and the world, there can be no substitute for the Bible; it is our only hope. And much more of the same sort has the learned divine written in his last book, which, no doubt, will be placed on sale with the Carter, Brothers, of New York, at one dollar. We wish we had room for further quotations from a book which is significantly dedicated to mothers and daughters, because "They who rock the cradle rule the world."

In the preceding pages* articles are found which have been written by different individuals, bearing more or less distinctly on the family relation, and the teachings which ought to be given therein in order to educate the child away from the influence of wrong desires, and inclinations, and passions, and lead him to the adoption of principles, and practices, and habits which are calculated to fit him for the performance of the highest duties which belong to a useful citizen; and also to implant in his mind an inflexible and just religious sentiment to shield him against temptation to wrong doing, and to those vicious practices which lead to a premature grave

The unhappiness of those whose religious training has been neglected, is a strong argument for every parent to implant into the mind of the child some fixed religious sentiment as a means of prevention from falling into a misanthoropical state of mind, ending in a disabelief of all religion, and finally sinking into the grave an outcast from society, as was the case of Thomas Paine. Reference has also been made to the kinds of books and periodicals which are best adapted to family training; and something, too, of the duties and the capabilities and influences which the wife may have in the household; and it would be well if every married lady reader should lay these pages down, more resolved than ever, to follow the example of the Hungarian wife, by endeavouring to make home attractive; not only in the examples to be set before the children, but in the conduct before the husband, in one point, in which there is a general want of attention in half the houses in the land,—a point to which the newly married should begin to give attention as a first and indispensable duty; and that is, the wisdom on the part of the wife of making a habitual effort to secure the affection and respect of her husband, in those attentions to her personal appearance, to which Madam H. evidently attached a high importance. Be assured, that a wife never gains, but always loses, in the estimation of her husband, when she allows herself to fall into the too common mistake of supposing that her inattentions to her dress and personal appearance in the presence of her family are not observed with silent disappointment and disapproval. A dowdy wife, with frowsly hair and slatterly dress, lolling in her chair reading a novel, or who comes creeping into the breakfast room, not more than half awake when the remainder of the family are nearly ready to leave the table, and drawls out some apology that she was never used to getting up early; such women are a disgrace to themselves and the mothers who bore them and can not be either loved or respected, even were they angels else. A slovenly wife isn't fit even for a drudge, for the lowest servant in the household ought to be tidy. These novel-reading wives who dress only for company or for the street, and not from an innate love for all that is pure, and tasteful, and refined, are a curse to families and to society in general. They perpetuate their delinquencies to generations after them, and they are they whose sons grow up for the gallows or the penitentiaries, and whose memories are execrated, as in the remarkable example which begins this article. But let not fathers lose sight of their responsibilities and duties; that they are the special head of the family, and that not only their example but their daily teachings should be such as will be calculated to impress the minds of their children with a love and an admiration for all that is just and generous and elevating in practice, as well as in theory; in conduct as well as in sentiment.

And let the reader compare the life services of the thief and murderer whose latest breath was expended in bitter reproof of the parents who never gave him any religious instruction, with the beautiful life and ending of one whose praises follow and who, from earliest years was brought up in the nurture and admonition of the Bible truths which, if heeded, not only give the "promise of the life that now is, and that which is to come," but give assurence of that wisdom of which it has been said that "Length of days is in her right hand, and in her left hand, riches and honor;" in other words, health and happiness for a long life-time.

